# Pharmacological Treatment of Arterial Hypertension in Children and Adolescents in Lithuania

**DOI:** 10.3390/ijerph192113949

**Published:** 2022-10-27

**Authors:** Eleonora Ivanova, Dovilė Ruzgienė, Karolis Ažukaitis, Augustina Jankauskienė

**Affiliations:** Faculty of Medicine, Vilnius University, 03101 Vilnius, Lithuania

**Keywords:** antihypertensive agents, hypertension, treatment rates, pediatric

## Abstract

The global prevalence of arterial hypertension (AH) in the pediatric population is increasing, but therapeutic approaches and the choice of the most suitable antihypertensive medications remains challenging. The study aimed to estimate the prevalence, treatment rates, and pharmacological treatment patterns of children and adolescents with AH in Lithuania during 2019 using real-world data. The study population consisted of citizens of Lithuania aged 0 to 17 years, who were diagnosed with AH according to the International Classification of Diseases (ICD). The analysis of reimbursed antihypertensive medication prescriptions was performed according to AH etiology and age. The overall prevalence of AH by diagnostic ICD codes in 2019 was 0.29%:0.24% for primary and 0.05% for secondary. Treatment rates were 39.8% for primary AH and 66.3% for secondary AH. Angiotensin-converting enzyme inhibitors (ACEi) were the most popular medications irrespective of the etiology of AH or age. Beta-blockers were in the second place and used more often in older children. Calcium channel blockers were the third and angiotensin receptor blockers were the fourth most frequent choices. Enalapril was the most popular agent in the ACEi group and metoprolol in the beta-blocker group. Nearly forty percent of Lithuanian children with primary AH receive pharmacological therapy compared to two-thirds with secondary AH. Although ACEi are the predominant class of antihypertensive medications, discordances with available guidelines are evident, particularly in the overuse of beta-blockers and underuse of diuretics.

## 1. Introduction

Childhood arterial hypertension (AH) is a major public health threat. Elevated blood pressure (BP) in childhood strongly predicts AH in adulthood [1,2,3]. Moreover, AH in the early years is associated with premature death and worse cardiovascular outcomes in adulthood [4,5,6]. Early signs of target organ damage (left ventricular hypertrophy, early vascular aging, and kidney injury) are already evident in children with AH and have been shown to track into adulthood [1,6]. Thus, the control of BP is aimed at preventing target organ damage development and, consequently, adverse cardiovascular outcomes in adult life. Importantly, the regression of target organ damage has been reported in children with AH with non-pharmacological and pharmacological interventions [7].

The study aimed to estimate the prevalence of AH, AH treatment rates, and individual antihypertensive medications prescription rates by analyzing real-world data from national registries in Lithuania during 2019.

## 2. Materials and Methods

We have performed a cross-sectional analysis using nationwide coded data from national registries. The details on the data retrieved from different registries are described further. 

### 2.1. Nationwide Prescriptions of Antihypertensive Medications

The Lithuanian healthcare system employs a compulsory health insurance system. The state-provided universal healthcare coverage includes the reimbursement of antihypertensive medication prescriptions to children. Accordingly, data about reimbursed antihypertensive medication prescriptions were obtained from the population-based electronic database from the Lithuanian National Health Insurance Fund (SVEIDRA) database for the year 2019. The following data were available: the number of unique patients who were prescribed at least one antihypertensive medication and the total number of individual medication prescriptions. 

The population consisted of citizens of Lithuania aged 0 to 17 years, who were diagnosed with AH and given reimbursed pharmacological therapy. The patients were divided into four age groups: 0–3, 4–7, 8–12, and 13–17 years. Specific medications were then categorized into groups by pharmacological classes: angiotensin-converting enzyme inhibitors (ACEi), angiotensin receptor blockers (ARBs), beta-blockers (BBs), calcium channel blockers (CCBs), and other (diuretics, imidazoline receptor agonists, alpha-blockers, combined ACEi, and diuretic).

Each prescription had an indication (primary diagnosis), which was divided based on the International Statistical Classification of Diseases (ICD) and Related Health Problems, Tenth Revision, Australian Modification (ICD-10-AM) into primary (I10) and secondary AH (I15). 

### 2.2. Prevalence of Coded AH Diagnosis

Data about the prevalence of AH were received from the National Institute of Hygiene based on ICD-10 coding: primary AH (I10) and secondary AH (I15) for the year 2019. In the year 2019, Lithuania had already adopted a national e-Health system; thus, the coded diagnosis data for the full population was collected and analyzed centrally. The data were available only for the age group of 0 to 17 years.

### 2.3. Population Size Data

The data about the total number of children living in Lithuania were obtained from the Lithuanian Statistic Department database.

## 3. Results

**Figure 1:**
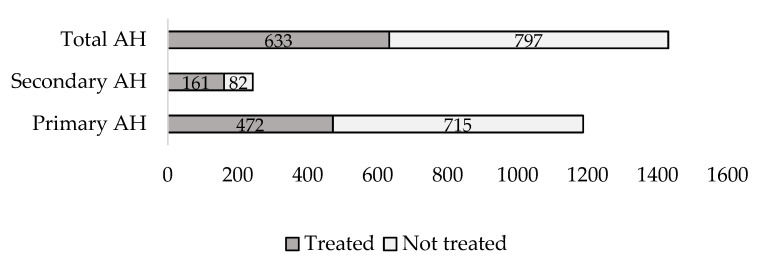
Number of patients diagnosed with AH and number of patients treated with pharmacological therapy by the etiology of AH.

There were 499,575 children (0 to 17 years) in Lithuania and 1430 children were diagnosed with AH in 2019: 1187 patients with primary AH and 243 with secondary AH. Accordingly, the prevalence of AH in 2019 was 0.29%:0.24% for primary AH and 0.05% for secondary AH. Medications were prescribed to 633 children in total and the treatment rates were 39.8% for primary hypertension and 66.3% for secondary hypertension (Figure 1).

**Figure 2:**
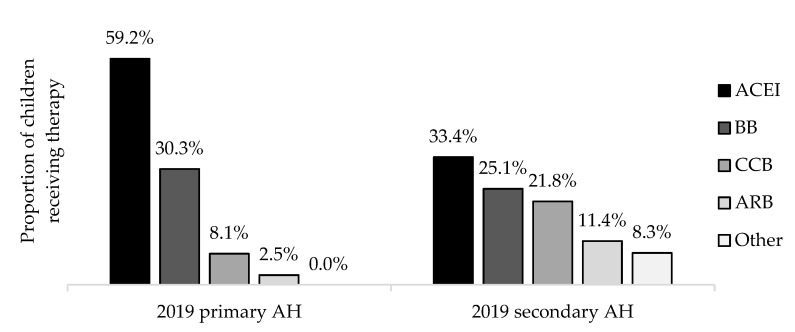
Distribution of drug classes used for the treatment by etiology of AH. Abbreviations: ACEi—angiotensin converting enzyme inhibitor, BB—beta blocker, CCB—calcium channel blocker, ARB—angiotensin receptor blocker.

A total of 1138 prescriptions were given out for the treatment of AH: 752 for primary AH and 386 for secondary AH. ACEi were the most frequently prescribed class of medications irrespective of AH etiology followed by BBs. CCBs and ARBs were the third and fourth most frequent choices. Other medication classes (diuretics, imidazoline receptor agonists, alpha-blockers, combined ACEi with diuretics) were only used in the treatment of secondary AH (Figure 2).

When stratifying by age groups and AH etiology, ACEi were the most frequently prescribed medications in primary AH patients irrespective of age, followed by BBs. Of note, CCBs that constituted the third most frequently prescribed medication class were only used in children older than 8 years of age, while the least prescribed ARBs were only used in 13- to 17-year-olds.

ACEi similarly were the most popular choice in the secondary AH group, except for children 8–12 years of age where BBs were prescribed most frequently. BBs were the second most frequent medications, except for young children 0–3 years where CCBs were used more frequently, though the sample size was low. In the remaining age groups, CCBs were the third choice, followed by ARBs (Table 1).

In the analysis of individual medications by their class, the following medications were the most popular: ACEi—enalapril, BBs—metoprolol, ARBs—losartan. In the CCBs class, lacidipine and amlodipine were prescribed at comparable rates. Details about individual medication prescriptions are summarized in Table 2.

## 4. Discussion

This study provides a snapshot of real-world data on pediatric primary and secondary AH rates and therapy in Lithuania during 2019. Our data confirms the shift of AH etiology with a predominance of primary hypertension over secondary AH. Children with primary AH received pharmacological therapy less frequently (40%) compared to secondary AH where two-thirds of the children were prescribed pharmacological treatment. Most children with AH in Lithuania are treated with ACEi irrespective of AH etiology or age. The second most popular AH medication class is BBs, more frequently prescribed to older children.

A meta-analysis reported the overall prevalence of AH in the pediatric population to be 4% while in Europe the prevalence ranges from 2.2% to 12.8% and even more (up to 27–47%) in overweight or obese children [8,9,10,11]. The calculated prevalence of pediatric hypertension in Lithuania was 0.29% for the ages 0–17 years old, being almost fivefold higher for primary AH (0.24%) compared to secondary AH (0.05%). Unfortunately, due to a lack of specific data, we could not study the prevalence of AH in relation to obesity in our population, but rates of obesity in Lithuania have been reported to be at 6.9% in 2020 [12].

The pediatric AH prevalence data in our analysis is significantly lower than that reported in previous studies. It is important to note that prevalence estimates in prior reports come from community-based screening studies while our estimate is based on the physician-diagnosed and reported ICD coding data. Thus, our finding may suggest that AH is underdiagnosed in Lithuanian children with at least more than ten-fold lower diagnosis rates compared to the average reported in the literature. This is in line with previous findings from a study in northeast Ohio that showed diagnosis rates to be at least four times lower compared to the actual prevalence [13]. On the other hand, our prevalence data is comparable to that of military health insurance data from the United States, which reported an overall prevalence of childhood AH at 2.6/1000 [14]. The discrepancies between AH rates between community-screening based data and diagnosis-based prevalence are most likely related to insufficient screening for high BP in the pediatric population. In addition, procedural aspects of BP measurements and the proper interpretation of BP results may also contribute to reduced diagnostic rates. These potential explanations, however, are speculative and cannot be proven based on our data.

The treatment of pediatric AH should start with population-based strategies, such as combating childhood obesity and sedentary lifestyles as well as non-pharmacologic management. Non-pharmacological management consists of weight loss, regular physical activity, and reduced sodium intake. The implementation of these strategies is recommended in all children with AH [15,16]. Non-pharmacological treatment is frequently initiated in children with non-severe primary AH without target organ damage, while these measures are usually insufficient in those with secondary AH [1,17]. AH treatment rates vary and are dependent on the region; global AH treatment rates in adults have been reported to be 38% for men and 47% for women [18].

The data on children is limited, but the previously mentioned military health care database analysis for the years 2006–2011 reported that 38.9% of children with AH received antihypertensive medications [14]. Our data showed similar treatment rates in children with a diagnosis of primary AH (39.8%), while treatment rates were significantly higher in children with secondary AH (66.3%). Children with secondary AH more frequently have more severe AH and thus exhibit signs of target organ damage; according to the European Society of Hypertension (ESH) guidelines, all cases of secondary AH require pharmacological therapy [1]. It is therefore interesting to note that only two-thirds of all the secondary AH population received antihypertensive medications. A meaningful interpretation of this finding, however, is limited due to missing patient-level data and details on secondary AH etiology and severity. However, in line with our findings, previous studies involving children with chronic kidney disease and coarctation of aorta have reported that up to one-fourth of children in these populations were either not receiving antihypertensive therapy or had uncontrolled AH [19].

The choice of pharmacological treatment for children with AH might be challenging due to limited evidence in the pediatric population. Thus, the guidelines have no definitive recommendations for the treatment as the evidence of antihypertensive therapy mostly relies on uncontrolled studies in heterogeneous populations [1,20]. Pediatric healthcare providers are frequently left with extrapolated dosing, safety, and efficacy from adult trials [21]. A 2014 Cochrane systematic review reported a lack of randomized trials, most of which did not have sufficient quality and were at risk of bias due to industry sponsoring [22]. Additionally, it is difficult to define endpoints for antihypertensive therapy studies as major cardiovascular events are rare in the pediatric age group [23]. Typically, the burden of AH in children is quantified by relying on surrogate markers of cardiovascular disease, such as left ventricular hypertrophy, carotid intima-media thickness, or albuminuria. Although scarcely studied, the regression of these markers has been observed with pharmacological and non-pharmacological therapies in the pediatric population [7,24,25].

Overall, ACEi, ARBs, CCBs, and diuretics are considered to be the acceptable first-line antihypertensive agents according to the available pediatric AH guidelines, while BBs are not recommended as first-line therapy by the American Academy of Pediatrics guidelines [1,20]. In line with previous studies, we have found that ACEi are the most common antihypertensive agents [14,26,27]. ACEi and ARBs are particularly suitable for patients with chronic kidney disease or proteinuric conditions due to their nephroprotective and antiproteinuric action and are generally considered a suitable choice for most children including those with primary AH [1]. The data from the US have similarly reported BBs and CCBs as the second and third most frequent medication classes [14]. However, compared to the US data, Lithuanian children were prescribed thiazides and alpha adrenergic receptor agents less frequently [14,27]. Particularly notable are the high rates of BB use in the Lithuanian population reaching 30% in those with primary AH compared to other populations where they constitute 10–20% of prescriptions [26,27]. Of note, BBs are prevalent in the treatment of adult hypertension in the Lithuanian population [28]. Thus, considering available guidelines, our data indicates the overuse of BBs and underuse of diuretics with a relatively low use of ARBs for the treatment of pediatric AH. It is difficult to speculate the reasons for BB overuse, but it might be related to the historical preference of BBs in Lithuania by adult physicians and the lack of familiarity with current ESH guidelines.

In the analysis of individual medication prescriptions, enalapril was the most popular choice from ACEi, metoprolol in BBs, while losartan predominated in the ARB group. All three medications went through randomized dose–response clinical trials in children and were effective and well-tolerated [29,30,31]. It is also important to note that our study revealed a couple of drugs that have not been mentioned in the 2016 ESH guideline: lacidipine and moxonidine. Both drugs lack pediatric evidence except for the retrospective analysis of lacidipine use in oncohematological patients and highlight the off-label use of AH medications in children [32]. It is possible that the use of these medications is determined by regional trends in the adult population as a 2019 study showed that moxonidine is more common as firs- and second-line therapy in Russia than in other countries [33].

Our study has several notable limitations: the coded data was provided for a total number of prescriptions and not on an individual level. Although it still represents medication selection patterns, it is unclear how long the therapy was prescribed and whether it needed to be changed. Additionally, there was no possibility to confirm the diagnosis and ensure no coding errors are present, as well as to evaluate concomitant medical conditions that could influence patient management. Finally, due to the nature of the study, we were unable to explore the reasons for antihypertensive medication selection in individual cases. On the other hand, our study’s strength is that all medications for pediatric AH management in Lithuania are reimbursed by universal health coverage and nationwide e-Health implementation allows us to gather an almost complete snapshot of the population.

## 5. Conclusions

Our data shows the predominance of primary AH in Lithuanian children, but relatively low diagnosis rates suggest that AH may be significantly underdiagnosed. ACEi are preferred antihypertensive agents for the treatment of primary and secondary AH across all age groups. On the other hand, our findings indicate the overuse of BBs and underuse of diuretics and ARBs and the undertreatment of children with secondary hypertension despite the available guidelines. In addition, several drugs lacking pediatric evidence are frequently used in children highlighting the off-label use of antihypertensive medications. Measures aiming to promote the adherence to existing pediatric AH guidelines are needed to improve the management of children with AH.

## Figures and Tables

**Table 1:** Rates of different class antihypertensive medication prescription by age and AH etiology.

AH Etiology	Age Group	ACEi (%)	BBs (%)	CCBs (%)	ARBs (%)	Other (%)
Primary AH	0–3 (*n =* 0)	-	-	-	-	-
	4–7 (*n =* 21)	85.7%	14.3%	-	-	-
	8–12 (*n =* 115)	68.7%	28.7%	2.6%	-	-
	13–17 (*n =* 616)	56.5%	31%	9.4%	3.1%	-
	All (*n =* 752)	59.2%	30.2%	8.1%	2.5%	-
Secondary AH	0–3 (*n =* 19)	47.4%	5.3%	15.8%	5.3%	26.2%
	4–7 (*n =* 63)	38.1%	27%	11.1%	17.5%	6.3%
	8–12 (*n =* 123)	27.6%	30.1%	27.6%	10.6%	4.1%
	13–17 (*n =* 181)	34.3%	23.2%	22.1%	10.5%	9.9%
	All (*n =* 386)	33.4%	25.1%	21.8%	11.4%	8.3%

AH—arterial hypertension, ACEi—angiotensin converting enzyme inhibitor, BBs—beta blockers, CCBs—calcium channel blockers, ARBs—angiotensin receptor blockers.

**Table 2:** Rates of different class antihypertensive medication prescription by age and AH etiology.

Class of Medication (Number of Prescriptions)	Medications (Number of Prescriptions)
ACEi (*n =* 574)	Captopril (*n =* 12) Enalapril (*n =* 421) Fosinopril (*n =* 92) Lisinopril (*n =* 43) Ramipril (*n =* 6)
BB (*n =* 324)	Atenolol (*n =* 28) Metoprolol (*n =* 293) Propranolol (*n =* 3)
CCB (*n =* 145)	Amlodipine (*n =* 65) Lacidipine (*n =* 74) Nifedipine (*n =* 6)
ARB (*n =* 63)	Losartan (*n =* 62) Valsartan (*n =* 1)
Other (*n =* 32)	Doxazosin (*n =* 10) Enalapril et hydrochlorothiazide (*n =* 16) Hydrochlorothiazide (*n =* 1) Moxonidine (*n =* 4) Spironolactone (*n =* 1)

ACEi—angiotensin converting enzyme inhibitor, BBs—beta blockers, CCBs—calcium channel blockers, ARBs—angiotensin receptor blockers.

## Data Availability

The data presented in this study are available on request from the corresponding author. The data are not publicly available due to restrictions from the Vilnius Regional Biomedical Research Ethics Committee.
